# Dataset on mice body weights and food intake following treatment with PG545

**DOI:** 10.1016/j.dib.2018.08.179

**Published:** 2018-09-06

**Authors:** Safa Kinaneh, Mohammad Agbaria, Niroz Abu-Saleh, Shadi Hamoud

**Affiliations:** a Department of Physiology, Rappaport Faculty of Medicine, Technion, Haifa, Israel; b Department of Internal Medicine A, Rambam Health Care Campus, Haifa, Israel; c Lipid Research Laboratory, Rappaport Faculty of Medicine, Technion, Haifa, Israel; d Department of Internal medicine E, Rambam Health Care Campus and Rappaport Faculty of Medicine, Haifa, Israel

## Abstract

This data article contains analysis of data observed in E_0_ mice placed on high fat diet, and treated by intraperitoneal injections of either normal saline (control) or the heparanase inhibitor PG545, in two different doses. Mice body weights and food intake were measured weekly and analyzed data are presented in graphs. Data will be of value for further understanding the role of the enzyme heparanase in controlling food intake and body weight. For further interpretations, see please “Heparanase inhibition attenuates atherosclerosis progression and liver steatosis in E_0_ mice” (Muhammad et al. 2018).

**Specification table**

**Table:** 

Subject area	*Medicine*
More specific subject area	*Metabolism*
Type of data	*Figure*
How the data were acquired	*Weighing mice body weights and food consumption weekly using a suitable scale and all graphs were obtained using GraphPad Prism 5.*
Data format	*Analyzed*
Experimental factors	*Mice were treated with weekly intra-peritoneal injections of either normal saline or PG545 for 12 weeks.*
Experimental features	*Mice body weights and food intake were assessed weekly and data presented in the attached figure.*
Data source location	*The Rappaport Faculty of Medicine, Technion, Israel institute of technology, Haifa, Israel.*
Data accessibility	*The data are in this article.*
Related research article	Shekh-Muhammad R, Abu-Saleh N, Kinaneh S, Agbaria M, Sabo E, Grajeda-Iglesias C, Volkova N, Hamoud S: Heparanase inhibition attenuates atherosclerosis progression and liver steatosis in E_**0**_ mice (in press). *Atherosclerosis* 2018 [1].

**Value of data**

● Data from a well-designed research, dealing with a common health concern with poor understanding and lacks efficient treatment options.
● The data provide a new insight into investigating and offering a possible therapeutic option for a common health concern.
● Treating weight gain or obesity is of therapeutic value in controlling several diseases, such as diabetes mellitus, hypertension, hyperlipidemia and many others.
● The data provide a basis for further research towards unveiling underlying mechanisms of obesity, dyslipidemias and related morbidity, and affording treatment options for such a common phenomenon.

## 1 Data

**Fig. 1:**
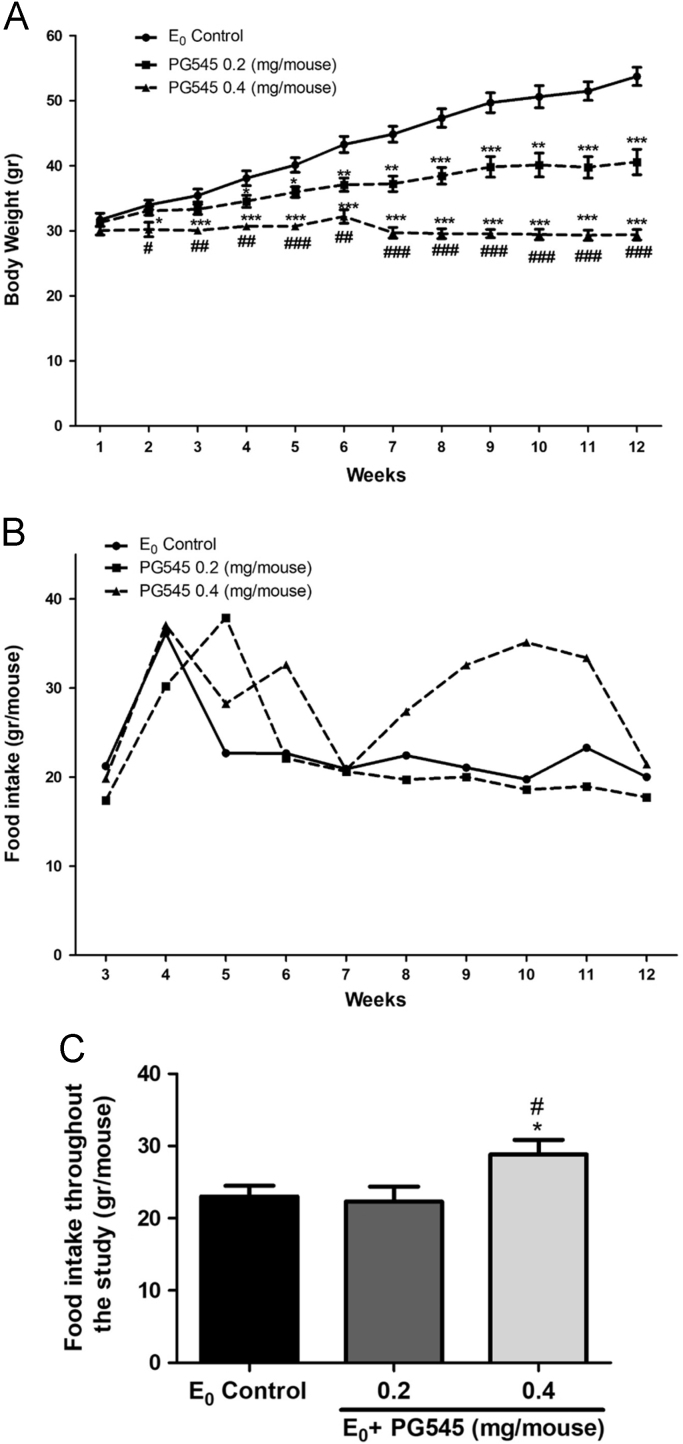
Effect of PG545 on mice body weight and food intake throughout the study in E_0_ mice**.** Weekly measurements of mice body weight (grams, A), food intake (per mouse in grams, B) and mean food intake throughout the study (grams/mouse, C). Values are presented as mean ± SEM. * Compared to control group. # Compared to PG545 low-dose group, */# *p* < 0.05, **/## *P* < 0.01, ***/### *P* < 0.001.

The data present the weekly measurements of the average mice body weights (in grams, Fig. 1A), weekly food intake (per mouse in grams, Fig. 1B) and mean food intake throughout the study period (per mouse in grams, Fig. 1C). Values are presented as mean ± SEM.

## 2 Experimental design, materials and methods

### 2.1 Animal studies

Male E_0_ mice, 12–13-week-old (Body weight ~30 g/mouse at baseline) were bred and housed in a pathogen-free environment and placed on high fat diet (HFD). The study was conducted according to the National Institutes of Health guideline and was approved by the Technion Ethics Committee (Ethics no. IL1090717).

### 2.2 Experimental design

Sham-Control group (*n* = 6) received weekly normal saline injections (0.1 ml/mouse, intraperitoneally – IP). Treatment groups (*n* = 7 in each) were treated with PG545 at either 0.2 mg/mouse (6.4 mg/kg − the low dose group) or 0.4 mg/mouse (13.3 mg/kg − the high dose group) administered IP once a week for 12 weeks [2], [3].

Mice body weights and food intake were assessed weekly. Data were analyzed and conducted using GraphPad Prism version 5.03 (GraphPad Software, Inc. CA, 92037 USA). A value of *p* < 0.05 was considered statistically significant. Data are presented as mean ± SEM.
