# Supplementary material for: Dataset on mice body weights and food intake following treatment with PG545
Source: Data Brief. 2018 Sep 6;20:1305–8. doi: 10.1016/j.dib.2018.08.179 (PMC6143748; doi:10.1016/j.dib.2018.08.179)
Supplement: Supplementary file 1 — Supplementary material [file mmc1.docx]

**Conflict of interest form**

**Hereby, all the authors declare that there is no conflict of interest regarding the publication of this manuscript.**
